# Protective Face Masks: Effect on the Oxygenation and Heart Rate Status of Oral Surgeons during Surgery

**DOI:** 10.3390/ijerph18052363

**Published:** 2021-02-28

**Authors:** Antonio Scarano, Francesco Inchingolo, Biagio Rapone, Felice Festa, Sergio Rexhep Tari, Felice Lorusso

**Affiliations:** 1 Department of Innovative Technologies in Medicine & Dentistry, University of Chieti-Pescara, Via dei Vestini, 31, 66100 Chieti, Italy; felice.festa@unich.it (F.F.); sergiotari@yahoo.it (S.R.T.); 2 Department of Interdisciplinary Medicine, University of Medicine Aldo Moro, 70124 Bari, Italy; francesco.inchingolo@uniba.it; 3 Department of Basic Medical Sciences, Neurosciences and Sense Organs, “Aldo Moro” University of Bari, 70121 Bari, Italy; biagiorapone79@gmail.com; 4 Department of Medical, Oral and Biotechnological Sciences, University of Chieti-Pescara, Via dei Vestini, 31, 66100 Chieti, Italy; felice.lorusso@unich.it

**Keywords:** SARS-CoV-2, COVID-19, N95, FFP2 respirators, surgical mask, protective face masks, personal protective equipment, severe acute respiratory syndrome-related coronavirus

## Abstract

Background: Safety in medical work requires eye protection, such as glasses, and protective facial masks (PFM) during clinical practice to prevent viral respiratory infections. The use of facial masks and other full personal protective equipment increases air flow resistance, facial skin temperature and physical discomfort. The aim of the present study was to measure surgeons’ oxygenation status and discomfort before and after their daily routine activities of oral interventions. Methods: 10 male voluntary dentists, specializing in oral surgery, and 10 male voluntary doctors in dentistry, participating in master’s courses in oral surgery in the Department of Oral Surgery of the University of Chieti, with mean age 29 ± 6 (27–35), were enrolled. This study was undertaken to investigate the effects of wearing a PFM on oxygenation status while the oral surgeons were actively working. Disposable sterile one-way surgical paper masks (Surgical Face Mask, Euronda, Italy) and FFP2 (Surgical Face Mask, Euronda, Italy) were used and the mask position covering the nose did not vary during the procedures. The FFP2 was covered by a surgical mask during surgical treatment. A pulse oximeter was used to measure the blood oximetry saturation during the study. Results: In all 20 surgeons wearing FFP2 covered by surgical masks, a reduction in arterial O_2_ saturation from around 97.5% before surgery to 94% after surgery was recorded with increase of heart rates. A shortness of breath and light-headedness/headaches were also noted. Conclusions: In conclusion, wearing an FFP2 covered by a surgical mask induces a reduction in circulating O_2_ concentrations without clinical relevance, while an increase of heart frequency and a sensation of shortness of breath, light-headedness/headaches were recorded.

## 1. Introduction

The global impact of the novel SARS-CoV-2 has had severe implications for dental healthcare providers. The safety of medical work requires an adequate use of facial protective equipment against droplet transfer of SARS-CoV-2 [1]. Eye protection such as glasses and protective facial masks are widely used in clinical practice to prevent viral respiratory infections. Introduced in the medical field by Mikulicz in 1897 and worn by surgeons and staff during medical treatment, with a change partway through long procedures [2,3]. Today, during pandemic influenza SARS-CoV-2, there is greater attention to the use of surgical masks. COVID-19, caused by a newly discovered coronavirus [4,5], has produced a quantitatively increased scientific production on the topic of COVID-19 [6,7,8]. This illness is a serious infection of respiratory system, especially in patients with underlying medical problems such as chronic respiratory disease, cardiovascular disease, cancer, diabetes and patient in hemodialysis. In these medical conditions, the patients develop severe acute pneumonia with a high percentage mortality rate [9]. In fact, around 20% of COVID-19 patients develop a critical or serious form of disease (Adult Respiratory Distress Syndrome), with a high percentage of cases (19–32%) that require respiratory support treatment [10]. It is an droplet-transmissible infection which can spread when an infected person talks, sneezes, coughs, or disperses mouth and nasal fomite secretions into the air [11]. Greater droplets may speedily settle on the surface or transmit disease to individuals in close proximity, while lesser droplets may remain suspended in the air for a long time and can contribute to transmission of the disease over great distances [12,13] and for prolonged time [14]. To reduce SARS-CoV-2 infection, many countries have adopted the obligation for medical workers to wear masks with superior filtering power, and, in many countries, FFP2 are being used, possibly covered by a surgical mask during medical treatment. The increase of air flow resistance, facial skin temperature and physical discomfort during wearing the PFM can induce the doctor to move this, with increasing risk of contagion [15]. However, clinical evidence is inadequate regarding whether SMs are less effective than N95 respirators for preventing viral respiratory infections, including influenza, in doctors and other healthcare workers [16]. Oral surgeons operating face-to-face with the patients are at high risk of catching respiratory infections [6]. During oral surgery procedures there is aerosol production [17,18], for this reason full personal protective equipment against respiratory infections must be worn. Usually physical distancing is recommended, but in dental work, dental hygienists and dental assistants are potentially in contact face-to-face with the patient, and, therefore, the use of an PFM is very important. A solution of 1% hydrogen peroxide or 0.2% povidone-iodine, as an antiseptic mouth rinse is recommended and should be used at the beginning of every treatment in dentistry to reduce the macrobiotic load and the viral one (SARS-CoV-2) in the saliva [19,20,21]. Moreover, essential procedures for the control and prevention of respiratory infections in dental clinic and healthcare environments are represented by surface cleaning and disinfection protocols [22]. Dental care cannot be stopped nor denied during the SARS-CoV-2 pandemic because urgent dental pathologies require immediate attention to help and reduce the burden on hospital emergency departments. Usually recommended are urgent dental treatments that are as minimally invasive as possible [19,23]. Dentists who wear personal protective equipment during oral surgery frequently experience fatigue, physical discomfort, and possibly even deterioration of surgical judgment and performance, despite the presence of standard air-conditioning in the operating rooms. This causes them to either wear the masks improperly or to remove them from the face. It has been suggested that the facial temperature augmentation discomfort is also caused by exhaled CO_2_ levels under the PFM, with sweating and hot flashes [15]. It appears reasonable that the increased CO_2_ levels under the PFM may also be trapped beneath them, causing a decrease in blood oxygenation. The aim of the present study was to measure the surgeons’ oxygenation status and discomfort before and after their daily routine activities of dental operations.

## 2. Materials and Methods

During the study period, May 2020 to October 2020, 10 male voluntary dentists, specializing in oral surgery, and 10 male voluntary doctors in dentistry, participating in a master program in oral surgery from our Department of Oral Surgery of the University of Chieti-Pescara, Italy with mean age 29 ± 6 (27–35), were enrolled. The study was carried out in observance of the Helsinki Declaration (revised version of Tokyo in 2004) and Good Clinical Practice Guidelines. All the surgeons signed informed consent for the adopted noninvasive procedure. The inclusion criteria were experience in oral surgery and the use of PFM. The primary exclusion criteria were presence of inflammation on the facial skin, lax skin, showing facial aging, allergic rhinitis and nasal septum deviations, facial treatment, including antiaging facial skin resurfacing, facial soft tissue augmentation or dermal filler, severe illness, facial skin disease, head and neck radiation therapy, chemotherapy, uncontrolled diabetes and beard. They had all previous experience in the use of a different type of PFM. In the previous hours, they had not experienced athletic training. The presence of respiratory diseases or smoking was exclusion criteria and none of the surgeons were overweight. After a thorough preliminary examination, the surgeons underwent pulse oximetry evaluation, being extensively informed concerning the study procedures. The surgeons entered a room with a constant temperature for 10 min to allow them to acclimatize. This research was undertaken to investigate the effects of wearing a PFM (FFP2) covered by surgical mask on oxygenation status while the oral surgeons were actively working. Disposable sterile one-way surgical paper masks (Surgical Face Mask, Euronda, Italy) and FFP2 (Surgical Face Mask, Euronda, Italy) were used and the mask position did not vary during the procedures (covering the nose). The FFP2 was covered by a surgical mask during surgical treatment.

The operations were grouped according to the duration:

- Duration of the operations was up to 20 min, (*n* = 25);
- Duration of the operation was between 20–40 min (*n* = 20);
- Duration of the operations was between 40–120 min (*n* = 15);
- Duration of the operations was between 120–240 min (*n* = 4).

In total, 64 surgeries were performed. After each intervention, the surgeons were invited to register, what they perceived, sensation of shortness of breath, light-headedness and headaches using visual analogic score (VAS) scale. All sensations were scored by means of a 100 mm VAS from 0 (no discomfort) to 100 (worst discomfort imaginable). A pulse oximeter with a recyclable clip type finger probe (Cardiocap/5, Datex-Ohmeda, Helsinki, Finland) was used to measure the blood oximetry saturation during the study. The Surgeons were encouraged to behave in their usual manner throughout the oral surgery. The finger probe was applied to the second finger of the right hand. Just before the operation, pulse rate and oxygen saturation values were recorded. At the end of the oral surgery, the pulse oximeter was utilized again, and the values were recorded. During this study, no surgeon developed the COVID-19 disease.

### Statistical Analysis

The research sample size was measured through a dedicated clinical software able to calculate the quantity of subjects needed to achieve statistical significance for quantitative analyses of hypoxemia and discomfort. The statistical model was performed for dichotomous variables (yes/no effect) according to the following parameters: incidence effect (85% for the Test group and 10% for the control group), alpha = 0.05 and power = 95%. The optimal research sample size was 20 surgeons. The study data were statistically evaluated by the software package Graphpad 8.0 (Prism, San Diego CA- USA). The normal distribution of the study data was evaluated by the Kolmogorov–Smirnov test. The descriptive statistics of all study variables was provided by means and standard deviations (SD) of all the experiments. The comparison of the groups on the research variables were evaluated by the two-way ANOVA test followed by post hoc Tukey HSD test. The one-way ANOVA followed by Tukey post hoc test was performed to evaluate the significance of the comparison between the study groups of shortness of breath and light-headedness/headache VAS score perception. The level of significance was set at *p* < 0.05.

## 3. Results

**Figure 1:**
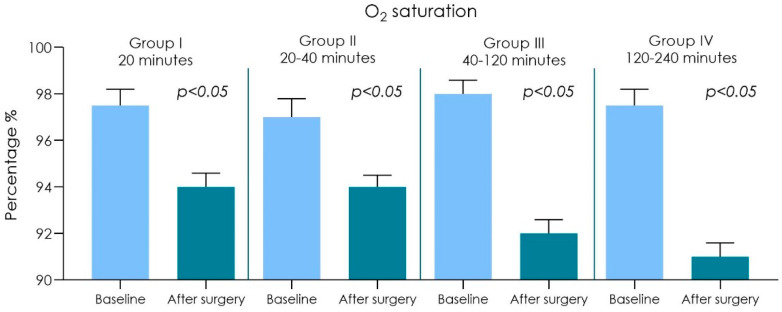
Graph chart of the surgeons O_2_ saturation (%) of Group I (20 min), II (20–40 min), III (40–120 min) and IV (120–240 min) (ANOVA post hoc Tukey HSD). No differences were detected between the baseline for both of Group I, II, III and IV. A statistically significant difference was detected in all comparisons after the surgery procedure (*p* < 0.05).

**Figure 2:**
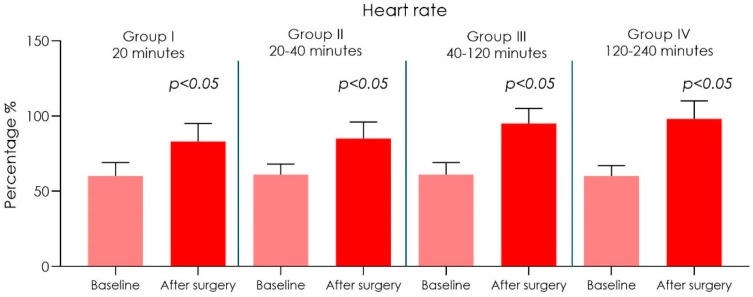
Graph chart of the surgeons’ heart rate (bmp) of Group I (20 min), II (20–40 min), III (40–120 min) and IV (120–240 min) (ANOVA post hoc Tukey HSD). No differences were detected between the baseline for both of Group I, II, III and IV. A statistically significant difference was detected by group comparison after the surgery procedure (*p* < 0.05).

In all 20 surgeons wearing FFP2 covered surgical masks a reduction in arterial O_2_ saturation was recorded from around 97.5% before surgery to 94% after surgery (Figure 1 and Table 1). Additionally, an increase in heart rate was also noted. The surgeons had before surgery heart rates of 60 ± 9 bpm beats/min, after surgery the dentists heart rates increased to 98 ± 12 bpm beats/min (range = 74–98; equivalent to 5–20% HRR). (Figure 2 and Table 1)

- Duration of the operations was up to 20 min (*n* = 25): an arterial O_2_ saturation was recorded from around 97.5% before surgery to 94% after surgery. Heart rate from 60 ± 9 bpm before surgery to 83 ± 12 bpm after surgery was also noted. Shortness of breath scored 30.33 ± 7.17 while light-headedness and headaches scored 21.33 ± 5.85.
- Duration of the operations was in between 20–40 min (*n* = 20): an arterial O_2_ saturation was recorded from around 97% before surgery to 94% after surgery. Heart rate from 61 ± 7 bpm before surgery to 85 ± 11 bpm after surgery was also noted. Shortness of breath scored 34.33 ± 6.91 while light-headedness and headaches scored 24.33 ± 5.11.
- Duration of the operations was in between 40–120 min (*n* = 15): an arterial O_2_ saturation was recorded from around 98% before surgery to 92% after surgery. Heart rate from 61 ± 8 bpm before surgery to 95 ± 10 bpm after surgery was also noted. Shortness of breath scored 35.33 ± 7.17 while light-headedness and headaches scored 28.33 ± 5.87.
- Duration of the operations was in between 120–240 min (*n* = 4): an arterial O_2_ saturation was recorded from around 97.5% before surgery to 91% after surgery. Heart rate from 60 ± 7 bpm before surgery to 98 ± 12 bpm after surgery was also noted. Shortness of breath scored 39.33 ± 7.64 while light-headedness and headaches scored 31.18 ± 4.85.

**Figure 3:**
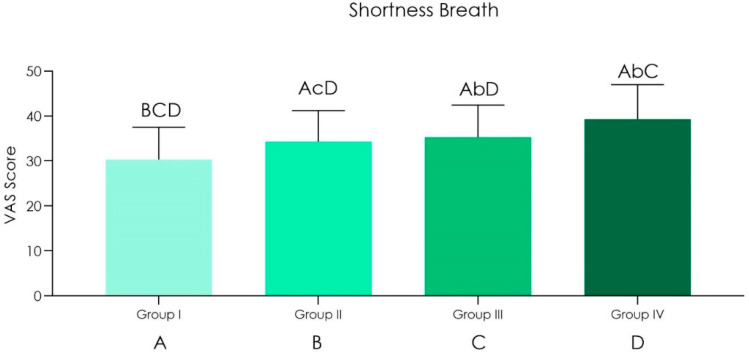
Graph chart of the shortness breath visual analogic score (VAS) score perception of Group I (20 min), II (20–40 min), III (40–120 min) and IV (120–240 min) (ANOVA post hoc Tukey HSD) (Top Bars between groups comparison: lower case *p* < 0.05; upper case: *p* > 0.05).

**Figure 4:**
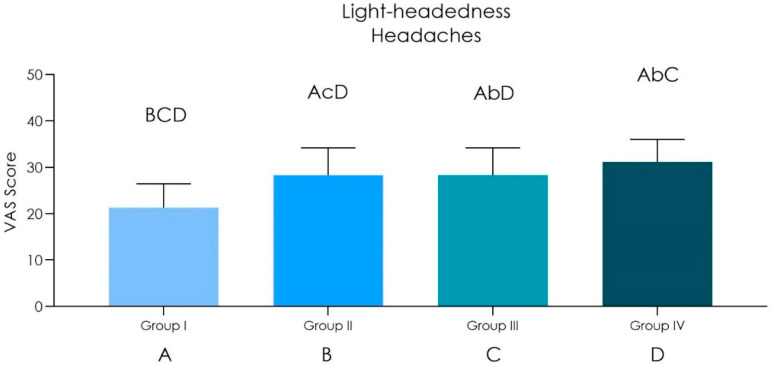
Graph chart of the light-headedness and headaches VAS score perception of Group I (20 min), II (20–40 min), III (40–120 min) and IV (120–240 min) (ANOVA post hoc Tukey HSD) [Top Bars between groups comparison: lower case *p* < 0.05; upper case: *p* > 0.05].

A statistical difference of oxygen saturation of hemoglobin was observed, between preoperational and post operational tests (Table 1 and Figure 1). We found a direct correlation between the increased duration of the operation and decrease of oxygen saturation of hemoglobin without it being significantly different, but it was observed that the heart pulse rate intensified after the surgical session (Figure 1 and Figure 2), and there was also a statistically significant difference in the sensations of shortness of breath, light-headedness and headaches increased with the time of surgery (Figure 3 and Figure 4 and Table 2).

## 4. Discussion

The present study results indicate that continuous wearing of Facial Masks during oral surgery led to a decrease in oxygen saturation of hemoglobin and an increase in heart rate. From the results, it looks like that the data show regular variation with the increase of operation time. Additionally, light-headedness and headaches and thermal discomfort were recorded. Normal blood O_2_ saturation is a fractional saturation of 90 to 97.5%, which corresponds to an arterial oxygen partial pressure of 13.3 to 13.7 kPa, if there are no other hemoglobin species, apart from oxygen reduced hemoglobin. However, the test has been carried out with reliability in most healthy young, individuals, while it has shown errors or failed to elicit signal in less healthy individuals due to several artifacts [24]. Pulse oximetry is a simple, noninvasive and standard method for monitoring a patient in use in operating rooms and care units for early detection of hypoxemia [25]. It is used for detecting the presence of hypoxemia and pulse oximeters may lead to a faster treatment of grave hypoxemia and possibly circumvent serious complications [26,27]. The device uses spectrophotometric technology, while the pulse oximetry is able to calculate the oxygen saturation by illuminating the finger skin through the changes of light absorption between the oxygenated and deoxygenated blood [28]. Reduced hemoglobin and oxygenated blood are able to absorb red and infrared light differently. The hemoglobin absorbs more red light while reduced oxyhemoglobin spectral absorption is characterized by more infrared light. The ratio of absorptions at the infrared and red wavelengths is measured by oximetry that indicates the oxygen saturation of arterial pulsations [29]. A face mask is able to prevent transpiration and give protection against airborne transmitted bacteria or virus. For these reasons, wearing a mask is necessary in healthcare situations, especially in case of a pandemic [13,16]. The use of PFM during oral surgery has been correlated with complaints of light-headedness, headaches, as well as an increase in the exertion sensation and perceived shortness of breath [30]. Headaches and perceived shortness of breath are also major symptoms recorded by Dentists when wearing an FFP2 covered by an FM during oral surgery. Similar results were recorded by people wearing a mask during SARS-CoV-2, although these symptom described above are not correlated with oxygen shortage [31]. Protective eyewear and prolonged wearing of N95, 6 h per day, produce in most subjects, the headaches [31]. Similar results were reported by Lim and co. [32] in most (healthcare workers 37%) during the (SARS-CoV-1) epidemic in 2003, involving a virus comparable to SARS-CoV-2 that impacts on the upper respiratory tract. Probably, the design of the masks, a tight fit in combination with tight elastic straps, produces pain behind the contact points on the face and the ears. Many factors were associated with headaches while wearing masks. In fact, the PFM induces inadequate hydration and inappropriate eating patterns to avoid touching hands or the face. FM was also associated to sleep deprivation and physical and emotional stress. Additionally, other factors can cause headaches and discomfort, such as facial itching, nasal bridge scarring [33], presence of acne [34], rash/irritation [35] and discomfort associated to augmentation of facial temperatures [15]. The use of PFM does not provoke important variations of oxygen and carbon dioxide concentrations in the blood in yang population, while there has been recorded discomfort related to the increased temperature of the skin induced by the facemask and the induced breathing resistance. Surgeon discomfort persists through increased irritation during oral surgery while wearing a mask. The outcome of the study demonstrates that the heart rate was influenced by wearing FFP2s covered by FMs, probably facial temperature, and subjective humidity influence heart rate increase discomfort. This data has been discussed in a previous study [15]. The perioral area is very important for thermoregulation of the body, in fact thermal stimulus to the surface around the mouth, nose and cheek regulates heat exchange from the respiratory tract, this data was also discussed in a previous publication [15]. A small reduction of oxygen stimulates the sympathetic nervous system with the consequence of an increased heart rate [36]. It was probable that the surgeons felt unfit, fatigued, and had headaches and overall discomfort due to this reason. Therefore, the increases in skin temperature and heart rate could induce substantial additional stress and might reduce work tolerance of the wearer. The FM produces an important change in humidity, microclimates and temperatures which have profound influences on heart rate and thermal stress and subjective perception of discomfort [15] then increase the dead space respiratory under the mask. However, based on the previously discussed results, sensations of shortness of breath, light-headedness and headaches are not caused by changes to O_2_ and CO_2_ balance. There are many issues around the use of PFM, in the present research we have evaluated only healthy surgeons. In fact severe disease allergy and asthma can pose a risk in wearing a mask [37]. It is necessary to also investigate the impact of wearing PFM in old subjects with chronic diseases. Interest in the control of heart rate by the baroreceptor system dates back to the work of Marey, who in 1859 demonstrated the inverse relation between arterial pressure and heart rate [38]. Sympathetic and parasympathetic nervous system activity in response to changes in arterial pressure has been studied [39,40,41]. The heart rate is determined by the Sinoatrial Node (SAN)—the pacemaker of the cardiac muscle. Experimental studies reported that the heart rate, wall tension and contractility (or the velocity of contraction) are considered the major factors of myocardial oxygen consumption (MVO_2_). Wearing an PFM has a high impact on the cardiopulmonary resuscitation efficacity, in fact it increases rescuer’s fatigue and reduces chest compression with more stringent requirements on the depth and frequency, thus compromising the rescue maneuvers [42]. N95 produces more rescuer’s fatigue during chest compression due to differences in vital signs including, respiration rate, mean heart rate, oxygen saturation and arterial pressure. This result indicates that an N95 mask induces discomfort in breathing with a typical reduction of 37% in air exchange volume [43]. A recent study found that when wearing an PFM there is evident markedly negative impact on exercise parameters, such as maximum oxygen uptake and the maximum power output, when wearing FFP2/N95 instead of a surgical mask [44]. In fact, the authors found that surgical masks and FFP2/N95 have a marked negative impact on the pulmonary parameters by 23% and of VO_2_max by 13% related to an increased airway resistance [43]. There are many studies that show that the use of PFM increases upper airway obstruction and a decrease of breathing frequency with corresponding variations of the exhaling and inhaling time and a reduced tidal volume [45,46,47]. Increased breathing resistance during wearing an PFM for a long period, especially FF2/N95 and FFP2 covered by a surgical mask, leads to increased work of the respiratory muscles with higher oxygen consumption, leading to negative augmentation of intrathoracic pressure (ITP). The increased negative intrathoracic pressure for long duration, increased to cardiac afterload, increases in enhanced myocardial oxygen consumption [48,49,50] with reduction of cardiac power by approximately 10%, which is compensated in healthy people. This compensation cannot be possible in patients with compromised myocardial function. These mechanisms induce sympathetically mediated vasoconstriction in the respiratory musculature with an increase of heart rate. Probably, the thermal discomfort and increased breathing resistance induce discomfort and stress with an increased heart rate. In fact, some studies found that the skin temperature increases effect heart rate and may cause substantial other types of stress and stimulation of the sympathetic nervous system in the wearer and might reduce work tolerance [36,51]. The decreased oxygenation in the study population can be explained by augmentation the dead space respiratory under the mask [52], increased breathing resistance with increased work of the respiratory muscles with higher oxygen consumption then increased heart rate with increases myocardial oxygen consumption. The observed elevated pulse rates, the light-headedness and headaches, increasing with the time of surgery, could be attributed to elevated pCO_2_ levels. In fact Anesthesiologists wearing a facial mask during percutaneous tracheostomy can experience dyspnea, tachycardia and tremor after 30 min [52]. No surgeons had a beard because facial hair is a major factor decreasing the protection of a FFP2 mask, as it prevents an adequate seal to be achieved [53]. In the present research we have not investigated the duration of use mask. The major limit of the present study was that there was no control group because it was conducted during the COVID-19 emergency. Another limit was that we have not investigated the effect of underlying medical conditions, age and sex as, in fact, the study was conducted on young healthy male surgeons. These young subjects might tolerate lower pO2 levels or might be more sensitive to changes in pCO2 levels. In the group IV there are only 4 subjects and this is another a limitation of this study. Further investigations with a larger numerosity and heterogenicity of the sample are required to clarify the study findings. In the present research we have not investigated on duration of use mask.

## 5. Conclusions

Two main conclusions emerge from this study: in healthy oral surgeons, wearing an FFP2 covered by a surgical mask for an extended period of time induces a reduction in circulating O_2_ concentrations without clinical relevance, while an increase of heart frequency and sensation shortness of breath, light-headedness and headaches were recorded. This result indicates that an N95 mask covered by surgical mask induce discomfort in breathing, decrease in both mental and physical performance, accuracy and increased fatigue, especially during lengthy operations, and could cause elevated pCO_2_ levels.

## Figures and Tables

**Table 1:** Summary of the surgeons O2 saturation (%) and heart rate (beats per minute-bmp) of Group I (20 min), II (20–40 min), III (40–120 min) and IV (120–240 min) (ANOVA post hoc Tukey HSD).

	Group I 20 min		Group II 20–40 min		Group III 40–120 min		Group IV 120–240 min	
	Baseline	After Surgery	Baseline	After Surgery	Baseline	After Surgery	Baseline	After Surgery
O_2_ saturation	97.5% ± 0.7	94% ± 0.6	97.0% ± 0.8	94% ± 0.5	98.0% ± 0.6	92% ± 0.5	97.5% ± 0.7	91% ± 0.6
*p* value	*p* < 0.05		*p* < 0.05		*p* < 0.01		*p* < 0.01	
Heart rate	60 ± 9 bpm	83 ± 12 bpm	61 ± 7 bpm	85 ± 11 bpm	61 ± 8 bpm	95 ± 10 bpm	60 ± 7 bpm	98 ± 12 bpm
*p* value	*p* < 0.01		*p* < 0.01		*p* < 0.01		*p* < 0.01	

**Table 2:** Summary of the surgeons visual analogic score (VAS) of shortness of breath and light-headedness and headaches of Group I (20 min), II (20–40 min), III (40–120 min) and IV (120–240 min) (ANOVA post hoc Tukey HSD).

VAS SCORE	Group I 20 min	Group II 20–40 min	Group III 40–120 min	Group IV 120–240 min
Short Breathness	30.33 ± 7.17	34.33 ± 6.91	35.33 ± 7.09	39.33 ± 7.64
Light-headedness- Headaches	21.33 ± 5.85	24.33 ± 5.11	28.33 ± 5.87	31.18 ± 4.85

## Data Availability

All experimental data to support the findings of this study are available contacting the corresponding author upon request. The authors have annotated the entire data building process and empirical techniques presented in the paper.

## References

[1] World Health Organization (2020). Rational Use of Personal Protective Equipment for Coronavirus Disease (COVID-19) and Considerations during Severe Shortages: Interim Guidance, 6 April 2020.

[2] Li Y., Tokura H., Guo Y.P., Wong A.S.W., Wong T., Chung J., Newton E. (2005). Effects of Wearing N95 and Surgical Facemasks on Heart Rate, Thermal Stress and Subjective Sensations. Int. Arch. Occup. Environ. Health.

[3] Romney M.G. (2001). Surgical Face Masks in the Operating Theatre: Re-Examining the Evidence. J. Hosp. Infect..

[4] Chen J. (2020). Pathogenicity and Transmissibility of 2019-NCoV-A Quick Overview and Comparison with Other Emerging Viruses. Microbes Infect..

[5] Zhu N., Zhang D., Wang W., Li X., Yang B., Song J., Zhao X., Huang B., Shi W., Lu R. (2020). A Novel Coronavirus from Patients with Pneumonia in China, 2019. N. Engl. J. Med..

[6] Bordea I.R., Xhajanka E., Candrea S., Bran S., Onișor F., Inchingolo A.D., Malcangi G., Pham V.H., Inchingolo A.M., Scarano A. (2020). Coronavirus (SARS-CoV-2) Pandemic: Future Challenges for Dental Practitioners. Microorganisms.

[7] Bellocchio L., Bordea I.R., Ballini A., Lorusso F., Hazballa D., Isacco C.G., Malcangi G., Inchingolo A.D., Dipalma G., Inchingolo F. (2020). Environmental Issues and Neurological Manifestations Associated with COVID-19 Pandemic: New Aspects of the Disease?. Int. J. Environ. Res. Public Health.

[8] Lorusso F., Inchingolo F., Scarano A. (2020). The impact of Covid-19 on the scientific production spread: A five-MONTH bibliometric report of the worldwide research community. Acta Med. Mediterr..

[9] Lee V.J., Aguilera X., Heymann D., Wilder-Smith A. (2020). Lancet Infectious Diseases Commission Preparedness for Emerging Epidemic Threats: A Lancet Infectious Diseases Commission. Lancet Infect. Dis..

[10] Critical care committee of Chinese Association of Chest Physician (2020). Conventional Respiratory Support Therapy for Severe Acute Respiratory Infections (SARI): Clinical Indications and Nosocomial Infection Prevention and Control. Zhonghua Jie He He Hu Xi Za Zhi.

[11] Kohanski M.A., Palmer J.N., Cohen N.A. (2020). Aerosol or Droplet: Critical Definitions in the COVID-19 Era. Int. Forum Allergy Rhinol..

[12] Xie X., Li Y., Sun H., Liu L. (2009). Exhaled Droplets Due to Talking and Coughing. J. R. Soc. Interface.

[13] MacIntyre C.R., Chughtai A.A., Rahman B., Peng Y., Zhang Y., Seale H., Wang X., Wang Q. (2017). The Efficacy of Medical Masks and Respirators against Respiratory Infection in Healthcare Workers. Influenza Other Respir. Viruses.

[14] Bourouiba L. (2020). Turbulent Gas Clouds and Respiratory Pathogen Emissions: Potential Implications for Reducing Transmission of COVID-19. JAMA.

[15] Scarano A., Inchingolo F., Lorusso F. (2020). Facial Skin Temperature and Discomfort When Wearing Protective Face Masks: Thermal Infrared Imaging Evaluation and Hands Moving the Mask. Int. J. Environ. Res. Public Health.

[16] Radonovich L.J., Simberkoff M.S., Bessesen M.T., Brown A.C., Cummings D.A.T., Gaydos C.A., Los J.G., Krosche A.E., Gibert C.L., Gorse G.J. (2019). N95 Respirators vs Medical Masks for Preventing Influenza Among Health Care Personnel: A Randomized Clinical Trial. JAMA.

[17] Scarano A., Carinci F., Lorusso F., Festa F., Bevilacqua L., Santos de Oliveira P., Maglione M. (2018). Ultrasonic vs Drill Implant Site Preparation: Post-Operative Pain Measurement Through VAS, Swelling and Crestal Bone Remodeling: A Randomized Clinical Study. Materials.

[18] Piattelli A., Piattelli M., Scarano A. (1997). Simultaneous Demonstration of Alkaline and Acid Phosphatase Activity in Bone, at Bone-Implant Interfaces and at the Epiphyseal Growth Plate in Plastic-Embedded Undemineralized Tissues. Biomaterials.

[19] ADA Develops Guidance on Dental Emergency, Nonemergency Care. Https://Www.Ada.Org/En/Publications/Ada-News/2020-Archive/March/Ada-Develops-Guidance-on-Dental-Emergency-Nonemergency-Care.

[20] Eggers M., Koburger-Janssen T., Eickmann M., Zorn J. (2018). In Vitro Bactericidal and Virucidal Efficacy of Povidone-Iodine Gargle/Mouthwash against Respiratory and Oral Tract Pathogens. Infect. Dis. Ther..

[21] Peng X., Xu X., Li Y., Cheng L., Zhou X., Ren B. (2020). Transmission Routes of 2019-NCoV and Controls in Dental Practice. Int. J. Oral Sci..

[22] Scarano A., Inchingolo F., Lorusso F. (2020). Environmental Disinfection of a Dental Clinic during the Covid-19 Pandemic: A Narrative Insight. Biomed. Res. Int..

[23] Dominiak M., Rózyło-Kalinowska IGedrange T., Konopka T., Hadzik J., Bednarz W., Matys Jacek Lella A., Rayad S., Maksymowicz R., Kuźniarski A. (2020). COVID-19 and Professional Dental Practice. The Polish Dental Association Working Group Recommendations for Procedures in Dental Office during an Increased Epidemiological Risk. J. Stomatol..

[24] Weber W.M., Elfadel I.M., Barker S.J. (1995). Low Perfusion-Resistant Pulse Oximetry. J. Clin. Monit..

[25] Place B. (1998). Pulse Oximetry in Adults. Nurs. Times.

[26] Jubran A., Tobin M.J. (1996). Monitoring during Mechanical Ventilation. Clin. Chest Med..

[27] Pretto J.J., Roebuck T., Beckert L., Hamilton G. (2014). Clinical Use of Pulse Oximetry: Official Guidelines from the T Horacic S Ociety of A Ustralia and N Ew Z Ealand. Respirology.

[28] Chan E.D., Chan M.M., Chan M.M. (2013). Pulse Oximetry: Understanding Its Basic Principles Facilitates Appreciation of Its Limitations. Respir. Med..

[29] Mayers J.R., Morgan E.G., Mikhail M.S., Murray M.J., Larson C.P. (2006). Patient Monitors. Clinical Anesthesiology.

[30] Rebmann T., Carrico R., Wang J. (2013). Physiologic and Other Effects and Compliance with Long-Term Respirator Use among Medical Intensive Care Unit Nurses. Am. J. Infect. Control.

[31] Ong J.J.Y., Bharatendu C., Goh Y., Tang J.Z.Y., Sooi K.W.X., Tan Y.L., Tan B.Y.Q., Teoh H.-L., Ong S.T., Allen D.M. (2020). Headaches Associated With Personal Protective Equipment—A Cross-Sectional Study Among Frontline Healthcare Workers During COVID-19. Headache.

[32] Lim E.C.H., Seet R.C.S., Lee K.-H., Wilder-Smith E.P.V., Chuah B.Y.S., Ong B.K.C. (2006). Headaches and the N95 Face-Mask amongst Healthcare Providers. Acta Neurol. Scand..

[33] Hu K., Fan J., Li X., Gou X., Li X., Zhou X. (2020). The Adverse Skin Reactions of Health Care Workers Using Personal Protective Equipment for COVID-19. Medicine.

[34] Tan K.T., Greaves M.W. (2004). N95 Acne. Int. J. Dermatol..

[35] Al Badri F.M. (2017). Surgical Mask Contact Dermatitis and Epidemiology of Contact Dermatitis in Healthcare Workers: Allergies in the Workplace. Curr. Allergy Clin. Immunol..

[36] Ganong W.F. (1995). Review of Medical Physiology.

[37] What People with Asthma Need to Know about Face Masks and Coverings during the COVID-19 Pandemic. Https://Community.Aafa.Org/Blog/What-People-with-Asthma-Need-to-Know-about-Facemasks-and-Coverings-during-the-Covid-19-Pandemic.

[38] Gronda E., Brambilla G., Seravalle G., Maloberti A., Cairo M., Costantino G., Lovett E., Vanoli E., Mancia G., Grassi G. (2016). Effects of Chronic Carotid Baroreceptor Activation on Arterial Stiffness in Severe Heart Failure. Clin. Res. Cardiol..

[39] Rosenblueth A., Freeman N. (1931). The Reciprocal Innervation in Reflex Changes of Heart Rate. Am. J. Physiol..

[40] Wustmann K., Kucera J.P., Scheffers I., Mohaupt M., Kroon A.A., de Leeuw P.W., Schmidli J., Allemann Y., Delacrétaz E. (2009). Effects of Chronic Baroreceptor Stimulation on the Autonomic Cardiovascular Regulation in Patients with Drug-Resistant Arterial Hypertension. Hypertension.

[41] Seravalle G., Dell’Oro R., Grassi G. (2019). Baroreflex Activation Therapy Systems: Current Status and Future Prospects. Expert Rev. Med Devices.

[42] Tian Y., Tu X., Zhou X., Yu J., Luo S., Ma L., Liu C., Zhao Y., Jin X. (2020). Wearing a N95 Mask Increases Rescuer’s Fatigue and Decreases Chest Compression Quality in Simulated Cardiopulmonary Resuscitation. Am. J. Emerg. Med..

[43] Lee H.P., Wang D.Y. (2011). Objective Assessment of Increase in Breathing Resistance of N95 Respirators on Human Subjects. Ann. Occup. Hyg..

[44] Fikenzer S., Uhe T., Lavall D., Rudolph U., Falz R., Busse M., Hepp P., Laufs U. (2020). Effects of Surgical and FFP2/N95 Face Masks on Cardiopulmonary Exercise Capacity. Clin. Res. Cardiol..

[45] Melissant C.F., Lammers J.W., Demedts M. (1998). Relationship between External Resistances, Lung Function Changes and Maximal Exercise Capacity. Eur. Respir. J..

[46] Shaw K., Butcher S., Ko J., Zello G.A., Chilibeck P.D. (2020). Wearing of Cloth or Disposable Surgical Face Masks Has No Effect on Vigorous Exercise Performance in Healthy Individuals. Int. J. Environ. Res. Public Health.

[47] Kyung S.Y., Kim Y., Hwang H., Park J.-W., Jeong S.H. (2020). Risks of N95 Face Mask Use in Subjects With COPD. Respir. Care.

[48] Convertino V.A., Cooke W.H., Lurie K.G. (2005). Inspiratory Resistance as a Potential Treatment for Orthostatic Intolerance and Hemorrhagic Shock. Aviat. Space Environ. Med..

[49] Ryan K.L., Cooke W.H., Rickards C.A., Lurie K.G., Convertino V.A. (2008). Breathing through an Inspiratory Threshold Device Improves Stroke Volume during Central Hypovolemia in Humans. J. Appl. Physiol..

[50] Cheyne W.S., Harper M.I., Gelinas J.C., Sasso J.P., Eves N.D. (2020). Mechanical Cardiopulmonary Interactions during Exercise in Health and Disease. J. Appl. Physiol..

[51] White M.K., Hodous T.K., Vercruyssen M. (1991). Effects of Thermal Environment and Chemical Protective Clothing on Work Tolerance, Physiological Responses, and Subjective Ratings. Ergonomics.

[52] Fletcher S.J., Clark M., Stanley P.J. (2006). Carbon Dioxide Re-Breathing with Close Fitting Face Respirator Masks. Anaesthesia.

[53] Germonpre P., Van Rompaey D., Balestra C. (2020). Evaluation of Protection Level, Respiratory Safety, and Practical Aspects of Commercially Available Snorkel Masks as Personal Protection Devices Against Aerosolized Contaminants and SARS-CoV2. Int. J. Environ. Res. Public Health.

